# Leveraging Subjective Parameters and Biomarkers in Machine Learning Models: The Feasibility of *lnc-IL7R* for Managing Emphysema Progression

**DOI:** 10.3390/diagnostics15091165

**Published:** 2025-05-03

**Authors:** Tzu-Tao Chen, Tzu-Yu Cheng, I-Jung Liu, Shu-Chuan Ho, Kang-Yun Lee, Huei-Tyng Huang, Po-Hao Feng, Kuan-Yuan Chen, Ching-Shan Luo, Chien-Hua Tseng, Yueh-His Chen, Arnab Majumdar, Cheng-Yu Tsai, Sheng-Ming Wu

**Affiliations:** 1 Division of Pulmonary Medicine, Department of Internal Medicine, Shuang Ho Hospital, Taipei Medical University, New Taipei City 23561, Taiwan; 2 Division of Pulmonary Medicine, Department of Internal Medicine, School of Medicine, College of Medicine, Taipei Medical University, Taipei 11031, Taiwan; 3 TMU Research Center for Thoracic Medicine, Taipei Medical University, Taipei 11031, Taiwan; 4 Graduate Institute of Clinical Medicine, College of Medicine, Taipei Medical University, Taipei 11031, Taiwan; 5 Division of Cardiovascular Surgery, Department of Surgery, Wan Fang Hospital, Taipei Medical University, Taipei 11696, Taiwan; 6 Division of Cardiology, Department of Internal Medicine, School of Medicine, College of Medicine, Taipei Medical University, Taipei 11031, Taiwan; 7 Cardiovascular Research Center, Wan Fang Hospital, Taipei Medical University, Taipei 11696, Taiwan; 8 Research Center of Sleep Medicine, College of Medicine, Taipei Medical University, Taipei 11031, Taiwan; 9 School of Respiratory Therapy, College of Medicine, Taipei Medical University, Taipei 11031, Taiwan; 10 Centre for Immunobiology, Blizard Institute, Queen Mary University of London, London WC1E 6BT, UK; 11 International PhD Program for Cell Therapy and Regeneration Medicine, College of Medicine, Taipei Medical University, Taipei 11031, Taiwan; 12 Department of Civil and Environmental Engineering, Imperial College London, London SW7 2AZ, UK; 13 School of Biomedical Engineering, College of Biomedical Engineering, Taipei Medical University, 250 Wuxing Street, Taipei 11031, Taiwan

**Keywords:** chronic obstructive pulmonary disease (COPD), emphysema, percentage of low attenuation area (LAA%), long non-coding interleukin-7 receptor α-subunit gene (*lnc-IL7R*), machine learning

## Abstract

**Background/Objectives:** Chronic obstructive pulmonary disease (COPD) remains a leading cause of death worldwide, with emphysema progression providing valuable insights into disease development. Clinical assessment approaches, including pulmonary function tests and high-resolution computed tomography, are limited by accessibility constraints and radiation exposure. This study, therefore, proposed an alternative approach by integrating the novel biomarker long non-coding interleukin-7 receptor α-subunit gene (*lnc-Il7R*), along with other easily accessible clinical and biochemical metrics, into machine learning (ML) models. **Methods:** This cohort study collected baseline characteristics, COPD Assessment Test (CAT) scores, and biochemical details from the enrolled participants. Associations with emphysema severity, defined by a low attenuation area percentage (LAA%) threshold of 15%, were evaluated using simple and multivariate-adjusted models. The dataset was then split into training and validation (80%) and test (20%) subsets. Five ML models were employed, with the best-performing model being further analyzed for feature importance. **Results:** The majority of participants were elderly males. Compared to the LAA% <15% group, the LAA% ≥15% group demonstrated a significantly higher body mass index (BMI), poor pulmonary function, and lower expression levels of *lnc-Il7R* (all *p* < 0.01). Fold changes in *lnc-IL7R* were strongly and negatively associated with LAA% (*p* < 0.01). The random forest (RF) model achieved the highest accuracy and area under the receiver operating characteristic curve (AUROC) across datasets. A feature importance analysis identified *lnc-IL7R* fold changes as the strongest predictor for emphysema classification (LAA% ≥15%), followed by CAT scores and BMI. **Conclusions:** Machine learning models incorporated accessible clinical and biochemical markers, particularly the novel biomarker *lnc-IL7R*, achieving classification accuracy and AUROC exceeding 75% in emphysema assessments. These findings offer promising opportunities for improving emphysema classification and COPD management.

## 1. Introduction

Chronic obstructive pulmonary disease (COPD) is characterized by persistent airflow limitations and obstructive ventilation patterns, primarily caused by exposure to harmful substances [1]. The global prevalence of COPD among individuals aged 30–79 years was estimated to be 10.3% (95% confidence interval (CI): 8.2–12.8%), corresponding to approximately 390 (95% CI: 312.6–487.9) million cases worldwide [2]. COPD significantly contributes to mortality rates, ranking as the third leading cause of death globally [3]. The COPD mortality rates are 14.0 and 6.4 per 100,000 for European men and women, respectively, whereas in the USA, the respective rates are relatively higher at 21.3 and 18.3 per 100,000 [4]. However, it was reported that approximately 70% of COPD patients worldwide are underdiagnosed, while an estimated 30–60% of patients are overdiagnosed [5]. Consequently, effective strategies for COPD screening and management are essential for further advancements.

The progression of COPD involves several pathological changes, including emphysema, which is characterized by enlarged distal airspaces and alveolar wall destruction [6]. Emphysema is significantly associated with increased mortality (hazard ratio (HR): 1.52; 95% CI: 1.20–1.91; *p* < 0.001) [7], and its progression exacerbates breathlessness, further elevating the mortality risk [8]. In addition to pulmonary function tests (e.g., spirometry) [9], high-resolution computed tomography (CT; HRCT) is considered an accurate and sensitive method for evaluating emphysema development [10]. Specifically, the low attenuation area percentage (LAA%), defined as the proportions of voxels with an attenuation density of ≤−950 Hounsfield units (HU), is a widely used HRCT-derived parameter for quantifying emphysema severity [11,12]. The LAA% has been used to assess COPD development (i.e., emphysema progression) with a threshold of 15% [13]. However, both pulmonary function tests and HRCT have several limitations, such as radiation exposure (especially in children and infants), limited accessibility in community settings, and high resource demands for both medical personnel and patients [14,15]. Various biomarkers have been identified as potential alternatives for assessing COPD progression [16,17]. A systematic review revealed that a two-fold increase in several biomarkers, for example, white blood cell (WBC) levels (HR: 2.07, 95% CI: 1.29–3.31), was associated with an increased risk of COPD severity and mortality [18]. Previous studies proposed a novel biomarker, the long non-coding interleukin-7 receptor α-subunit gene (*lnc-IL7R*), which showed an inverse relationship with COPD risks. Specifically, decreased *lnc-IL7R* expression was associated with an increased probability of COPD [19,20]. However, these investigations were primarily observational or exploratory in nature and did not evaluate the predictive utility of the biomarker within a clinically integrated framework. Consequently, the potential role of *lnc-IL7R* in supporting COPD management remains uncertain. Additionally, variables such as demographic characteristics, body mass index (BMI), and subjective questionnaire outcomes may also serve as predictors of COPD development and progression [21,22]. Thus, a comprehensive approach integrating the aforementioned parameters may improve COPD management.

Machine learning (ML), with its ability to handle large datasets, is increasingly being applied in healthcare for disease prediction, diagnosis, and telemonitoring [23]. Although deep learning methods are capable of processing more complex data types, previous studies have demonstrated that they do not consistently outperform conventional machine learning approaches in the predictive assessment of COPD, particularly when applied to relatively small feature sets or limited datasets [24]. For example, a related study employed random forest (RF) models incorporating baseline demographics, family history, CT imaging features, and self-reported comorbidities to predict declines in the forced expiratory volume in 1 s (FEV_1_), an indicator of COPD development [25]. Given the resource-intensive nature of HRCT and its derived markers (e.g., LAA%), ML models leveraging accessible clinical and biochemical parameters may offer a promising alternative for COPD assessment. For instance, another study employed conventional ML algorithms with demographics and blood biomarker data (e.g., WBCs and platelet counts) to predict COPD progression and mortality risk, achieving area under the receiver operating characteristic curve (AUROC) values above 0.8 [26]. Despite these findings, the integration of conventional ML approaches with multidimensional clinical and biomarker data in COPD management remains poorly explored and warrants further investigation.

In this study, we evaluated the feasibility of using ML models with easily accessible data to comprehensively assess COPD development. A multimodal approach integrating baseline characteristics, a subjective questionnaire (COPD Assessment Test, CAT), and biochemical details was employed to classify the emphysema severity (LAA% <15% vs. LAA% ≥15%) in the COPD participants. Quantitative analyses were conducted to compare these groups, and the feature importance values were evaluated to identify key factors in emphysema progression (LAA% ≥15%). The findings may shed a light on the potential of leveraging ML models with multidimensional data to enhance COPD management in clinical settings.

## 2. Methods

### 2.1. Ethical Approval

This single-center, prospective, non-randomized cohort study was approved by the Joint Institutional Review Board of Taipei Medical University (TMU-JIRB approval nos. N201902021, N201502024, and N201803059, February 2015). All procedures and subsequent experiments followed the Declaration of Helsinki guidelines and the approved protocol. Informed consent was obtained from all participants prior to sample collection. Subsequently, data analyses were performed in accordance with the relevant guidelines and regulations.

### 2.2. Study Design and Patients

The participants were recruited from COPD cohorts regularly followed up at the Department of Thoracic Medicine, Taipei Medical University–Shuang Ho Hospital (New Taipei City, Taiwan) from April 2015 to February 2021. Firstly, pulmonary function tests and HRCT examinations were conducted to assess lung function and image attenuation values. Notably, the participants were excluded if they showed reversible airflow obstruction of >12% and 200 mL after bronchodilator inhalation (in accordance with American Thoracic Society guidelines), had a documented clinical history of previous or current asthma episodes, or exhibited coexisting abnormalities from HRCT. Only patients in a stable condition without acute exacerbations in the previous 3 months, no need for antibiotic or oral corticosteroid therapy, and no changes in respiratory symptoms were enrolled. Blood samples were collected from the eligible participants to obtain peripheral blood mononuclear cells (PBMCs) and plasma specimens and identify biochemical details. Subsequently, CAT was administered, and baseline characteristics and background information (smoking status and comorbidities) were retrieved from electronic clinical records. The Charlson comorbidity index was then calculated [27]. All data were prospectively collected and regularly followed up by a study nurse to ensure completeness and to avoid missing data.

### 2.3. HRCT Procedure and LAA Determinations

HRCT scans were performed using a GE Discovery CT 750 HD (GE, Fort Myers, FL, USA), with a 10 mm slice thickness. To ensure high-quality images, the participants were asked to take a deep breath and hold it during the lung scan. The derived serial images were analyzed using the commercial APOLLO workstation (version 1.2; VIDA Diagnostics, Coralville, IA, USA), which served as a centralized reading facility. Two specialized radiologists employed this system to determine the LAA% using a threshold of −950 HU [28]. The participants were then divided into two groups according to the LAA% (LAA% <15% and LAA% ≥15%) for further analysis [29,30].

### 2.4. lnc-IL7R Determinations

A reverse-transcription quantitative polymerase chain reaction (RT-qPCR) was used to determine the *lnc-IL7R* levels. The total RNA was extracted from PBMCs, and its concentration was determined using a NanoDrop ND1000 spectrophotometer (Nyxor Biotech, Paris, France). PCRs were prepared with the SYBR™ Green PCR Master Mix (cat. no. 4309155; Applied Biosystems, Waltham, MA, USA), specific primers, a fluorogenic probe mix, and the TaqMan Universal PCR Master Mix (Applied Biosystems, Waltham, MA, USA). Next, amplifications were performed in quadruplicate from 20 ng of complementary (c)DNA on a Bio-Rad C1000 real-time PCR system (Bio-Rad, Cambridge, MA, USA). The expression levels were normalized to 18S ribosomal (r)RNA (cellular) or GAPDH (extracellular). All procedures were strictly performed according to the protocol described in a previous study [31].

### 2.5. Statistical Analysis

All statistical analyses were performed using the open-source Python library Scikit-learn (version 0.21.2; Python Software Foundation, Fredericksburg, VA, USA). The Shapiro−Wilk test was initially applied to assess the normality of continuous variables. Based on the results, a Student’s *t*-test was used for variables with a normal distribution (*p* > 0.05), while the Whitney U-test was employed for non-normally distributed variables (*p* < 0.05). A Fisher’s exact test was conducted to identify differences between two groups for categorical variables. Next, linear regression models were applied to explore relationships between LAA% and biochemical details using both simple (without adjustment) and multivariable (adjusted for age, sex, and BMI) models. The results of the regression models are presented as beta coefficients with 95% CIs. The statistical significance was set to *p* < 0.05 for all tests. As a supplementary analysis, multivariable logistic regression was performed to assess whether body profiles (e.g., BMI) differed between groups independent of age, gender, and smoking status. To enhance statistical robustness, Cohen’s d was also calculated for selected biomarkers to evaluate the effect sizes.

### 2.6. ML and Feature Importance Values

To develop ML models for emphysema classification (LAA% ≥15%), this study considered both subjective and objective variables, including advanced biomarkers (e.g., *lnc-IL7R*), baseline characteristics, and CAT scores. These parameters were selected heuristically based on clinical experience and a review of the literature regarding their known relevance to COPD progression. Given the limited number of variables and the absence of imaging data, conventional ML approaches were chosen over deep learning to ensure model interpretability. Five ML models, encompassing support vector machine (SVM), k-nearest neighbors (kNNs), RF, naïve Bayes (NB), and logistic regression (LR), were applied for model development. Firstly, the dataset was systematically split into training and validation (80%) and test (20%) subsets. During the training phase, a meticulous 5-fold cross-validation grid search was performed to ensure robustness and generalizability. For the SVM model, the optimization focused on the kernel function, kernel coefficient Υ, and penalty parameter C. The RF model was optimized for the classification and regression tree, which was set to 250 [32], while the kNN model was tuned based on the number of neighbors and the weighting types. Next, the model performance was evaluated using metrics including accuracy, precision, recall, F1-score, and AUROC in both the training and test phases [33]. For the testing phase, bootstrapping techniques were applied to evaluate the model’s robustness comprehensively with confidence intervals.

The model with the highest accuracy and AUROC on the training dataset was selected for a further feature importance analysis. Additionally, feature importance was determined using the mean decrease in accuracy technique, with results visually represented in a bar chart. To elaborate, the mean decrease in accuracy computes feature importances based on the mean reduction in the model accuracy following random shuffling of feature values across the samples [34].

## 3. Results

### 3.1. Comparisons of Baseline Characteristics

Baseline characteristics of the COPD cohort (*n* = 80) were analyzed by stratifying the participants into two groups: LAA% <15% (*n* = 46) and LAA% ≥15% (*n* = 34) (Table 1). The mean ages were 67.02 (standard deviation (SD) = 7.65) and 68.44 (SD = 7.12) years for the respective groups, and the majority in both groups was male. The LAA% ≥15% group demonstrated a significantly smaller BMI (LAA% <15%: 24.91 ± 4.27 kg/m^2^; LAA% ≥15%: 21.24 ± 3.35 kg/m^2^, *p* < 0.01). The prevalence of comorbidities did not show significant differences. The primary COPD cohorts in both groups were either current smokers or had high smoking pack years (LAA% <15%: 95.64%, smoking pack years: 53.5 ± 36.85; LAA% ≥15%: 100%; smoking pack years: 63.93 ± 35.34). In the Supplementary Materials, the logistic regression results demonstrated body profile differences between the groups, accounting for confounders (e.g., age, gender, and smoking).

### 3.2. Comparisons of Pulmonary Function and Biochemical Details

Table 2 presents comparisons of pulmonary function measures and biochemical details between the two groups. Both groups exhibited abnormal pulmonary function measures characteristic of COPD (i.e., declined and impaired lung function). The FEV_1_ as a percentage of predicted was greater in the LAA% <15% group (64.53% ± 19.87%) than in the LAA% ≥15% group (50.25% ± 20.73%, *p* < 0.01). The ratio of FEV_1_ to forced vital capacity (FEV_1_/FVC) also significantly differed between the groups, with the LAA% <15% cohort demonstrating a ratio of 60.03% ± 10.32% compared to 51.67% ± 10.96% in the LAA% ≥15% group (*p* < 0.01). Regarding biochemical details, WBC and red blood cell (RBC) counts showed no significant differences between the groups. Neutrophil, lymphocyte, and eosinophil counts were also similar across the groups. Notably, the levels of *lnc-IL7R* differed significantly between the two groups, with mean values of 0.57 ± 0.25-fold in the LAA% <15% group and 0.43 ± 0.22-fold in the LAA% ≥15% group (*p* = 0.01). Furthermore, the Supplementary Materials document the Cohen’s d value for the *lnc-IL7R* difference between the two groups.

### 3.3. Exploring Links Between Lung CT Features and Biochemical Details

Associations between total lung LAA% derived from HRCT scans and biochemical parameters are presented in Table 3. The higher values of the LAA% were positively associated with WBCs and RBCs but did not reach statistical significance. Regarding the types of WBCs, higher values of the LAA% were positively associated with neutrophils but did not reach statistical significance. For *lnc-IL7R*, the values of the LAA% were significantly associated with a 2.93-fold decrease (95% CI: −4.85 to −1.01) in the crude models and a 2.65-fold decrease (95% CI: −4.33 to −0.97) in the multivariable models (adjusted for age, sex, and BMI).

### 3.4. Model Performance and Feature Importance in Predicting LAA Thresholds

**Figure 1:**
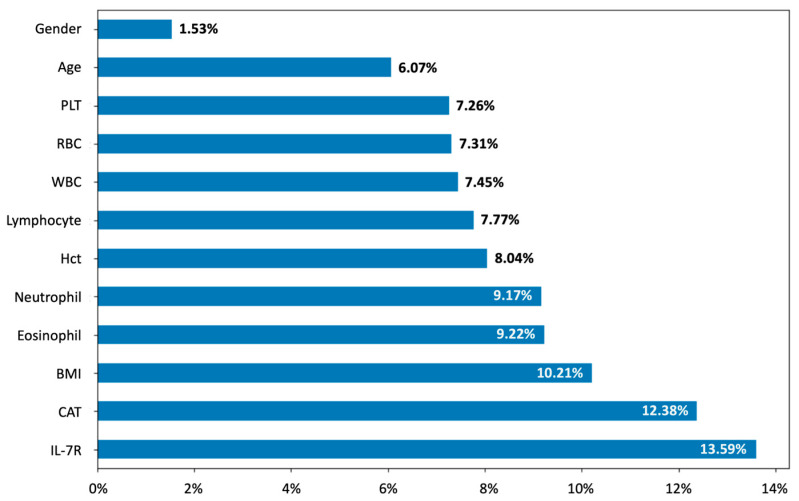
Feature importance of the selected model—random forest (RF)—for the test dataset.

The performance of the ML algorithms across the different phases is summarized in Table 4 (training and validation) and Table 5 (testing). During the training and validation phase (cross-validation outcomes, *n* = 64), the RF model demonstrated the highest mean accuracy of 75.15% ± 12.23% compared to the other models (LR: 65.62% ± 3.12%; kNN: 62.48% ± 7.82%; NB: 73.59% ± 12.29%; and SVM: 72.05% ± 7.45%). Similarly, the RF model exhibited the highest mean AUROC at 78.31% ± 5.91% compared to other models (LR: 74.09% ± 5.26%; kNN: 63.78% ± 8.39%; NB: 75.6% ± 5.31%; and SVM: 74.68% ± 8.84%). Subsequently, in the test phase (*n* = 16), the RF model demonstrated the highest mean accuracy of 75.31% ± 11.12% compared to the other models (LR: 69.38% ± 12.98%; kNN: 62.76% ± 11.97%; NB: 73.44% ± 1.56%; and SVM: 72.42% ± 15.4%). For the AUROC, the RF model also exhibited the highest value at 76.71% compared to the other models (LR: 69.65% ± 12.07%; kNN: 51.19% ± 11.64%; NB: 73.51% ± 20.78%; and SVM: 72.92% ± 17.49%). Regarding the feature importance of input variables, the fold change in *lnc-IL7R* presented the highest predictive value for the 15% LAA% threshold (Figure 1). The CAT scores and BMI values, respectively, showed the second- and third-highest importance values (CAT: 12.38%; BMI: 10.21%). As for the biochemical details, the levels of eosinophils and neutrophils, respectively, ranked as the fourth and fifth most important indicators (eosinophils: 9.22%; neutrophils: 9.17%).

The dataset was divided into 80% training/validation and 20% test subsets. The RF model, which achieved the highest accuracy and area under the receiver operating characteristic curve (AUROC) on the training/validation dataset compared to other models, was selected for a feature importance analysis on the test dataset. All parameters used in developing the machine learning models were analyzed. Abbreviations: PLT, platelet; RBC, red blood cell; WBC, white blood cell; HCT, hematocrit; BMI, body mass index; CAT, Chronic Obstructive Pulmonary Disease Assessment Test; *lncIL-7R*, long non-coding interleukin-7 receptor α-subunit gene.

## 4. Discussion

COPD is a major global health concern and a leading cause of death. Establishing an effective alternative method for emphysema classification, a key subtype of COPD, is essential to improve the overall disease management. While integrating multiple biomarkers offers advantages over individual ones [35], the feasibility of an ML model incorporating multidimensional parameters for classifying emphysema progression remains unclear. Hence, in this study, we collected baseline characteristics, CAT scores, and biomarkers from 80 participants to develop ML models for LAA% classification. BMI, *lnc-IL7R*, and pulmonary function measures significantly differed between the LAA% <15% and LAA% ≥15% groups. The fold change in *lnc-IL7R* showed significant negative associations with the LAA% in both the simple and multivariate-adjusted models, aligning with previous findings [36]. The RF model achieved the highest accuracy and AUROC compared to the other models and was chosen for the feature importance analysis. *lnc-IL7R* had the highest importance value on the LAA% classification, followed by CAT and BMI.

The majority of participants in this study were elderly (aged > 65 years) and were either current or ex-smokers. Previous studies identified age and smoking status as risk factors for COPD development. A cohort study in northern Sweden reported that the COPD prevalence was strongly influenced by smoking, with approximately 50% of smokers developing COPD, which significantly increased with age [37]. Similarly, another study found that an increasing age was correlated with higher COPD exacerbation rates, comorbidity prevalence, and mortality [38]. Declines in lung and immune functions, along with chronic inflammation [39,40], may account for the impact of aging on COPD development [41]. Additionally, mean BMI values were significantly lower in the LAA% ≥15% group than in the LAA% <15% group. A retrospective study found that a higher BMI was positively correlated with pulmonary function test variables (e.g., FEV_1_ and FVC), suggesting that individuals with a higher BMI tended to have better pulmonary function [42]. Another study revealed a U-shaped relationship between BMI and COPD mortality, with higher mortality observed when the BMI was either below 21 kg/m^2^ or above 35.25 kg/m^2^ [43]. Despite obesity being a major risk factor for various diseases [44], overweight and obese COPD patients have shown lower mortality rates—a phenomenon known as the obesity paradox [45,46]. The baseline characteristics of the participants in this study were consistent with previous findings, emphasizing the influence of these parameters on COPD development.

Regarding pulmonary function, the LAA% ≥15% group demonstrated lower values of FEV_1_ % predicted and the FEV_1_/FVC ratio compared to the LAA% <15% group, indicating a decline in lung functions with emphysema progression (higher LAA). The LAA% from HRCT scans reflects the alveolar lesion area associated with COPD development, which may explain these findings [47]. One study found that emphysematous individuals with a higher LAA% experienced more rapid declines in pulmonary functions compared to those with a lower LAA% [48]. Similarly, previous research showed an inverse relationship between pulmonary function measures and LAA%, further supporting LAA% as a reliable indicator of COPD development [49]. Additionally, *lnc-IL7R* showed a strong association with LAA%, and the values significantly differed between the LAA% <15% and LAA% ≥15% groups. The underlying mechanisms may involve interactions between inflammation and immune responses with COPD development. A previous study indicated that *lncRNA*s (e.g., *lnc-IL7R*) play a role in regulating inflammation—a key driver of COPD progression [50]. To be specific, knockdown of *lnc-IL7R* was associated with an increased expression of proinflammatory mediators, further supporting its role in inflammatory regulation [51]. Another study reported that *lnc-IL7R* was linked to immune regulation, with lower levels contributing to the inflammatory milieu in smokers’ lungs [52]. Taken together, these findings suggest that *lnc-IL7R* could serve as a promising biomarker for classifying emphysema progression.

The current models integrated both subjective and objective features to classify emphysema progression, including CAT scores, baseline characteristics, and biomarkers. Compared to traditional statistical methods, ML is more effective in handling large, complex data, which are common in the medical field [53]. Wang et al. developed ML models for identifying acute COPD exacerbation, which exhibited robust AUROC performances and underscored the potential of ML for COPD clinical diagnoses [54]. Among the models developed in this study, the RF model achieved the highest accuracy and AUROC in both the training and test datasets, followed by NB and SVM. This superior performance of the RF model may be attributed to its robustness and noise tolerance, which help prevent overfitting through the bootstrap method [55]. Owing to these strengths, the RF model has been widely applied in diagnostic and classification tasks in the medical field [56]. Considering the limited sample size, the simplicity and the lower risk of overfitting may explain the better performance of NB compared to SVM.

Regarding the feature importance of using the RF model on the test dataset, *lnc-IL7R*, CAT scores, and BMI were the strongest predictors for classifying LAA% <15% and LAA% ≥15%. As aforementioned, *lnc-IL7R* and BMI play significant roles in COPD development, consistent with current findings. Specifically, the advanced biomarker *lnc-IL7R* emerged as a strong indicator of COPD development, while BMI was correlated with pulmonary functions. The CAT questionnaire is a simple and reliable tool for evaluating the health-related quality of life in COPD patients [57]. CAT scores are linked to disease progression, with lower scores indicating recovery and symptom alleviation after treatment [58]. A retrospective study identified CAT scores as influential predictors of first-time acute exacerbation, aligning with the present findings [59].Overall, these results highlight the potential of incorporating these easily accessible variables into predictive models for improving COPD management.

To the best of our knowledge, this is the first study to establish ML models for emphysema classification incorporating both subjective (CAT scores) and objective (baseline characteristics and biomarkers) parameters. Notably, the present findings reinforce the feasibility of integrating the *lnc-IL7R* novel biomarker into model development. In addition, the feature importance analysis illuminated the impacts of these aforementioned parameters on classifying LAA% <15% and LAA% ≥15%, thereby aiding clinical interpretations. While radiation exposure and long waiting times hinder COPD management, the current models provide an alternative approach with easily accessible variables to improve efficacy and expand applications in community settings.

There are several limitations of this study that should be addressed in future research. Firstly, although the global COPD prevalence was higher in men (14.3%; CI: 13.3–15.3%) than in women (7.6%; CI: 7.0–8.2%) and increased with age [60], the high proportions of elderly male participants in this study may have limited the generalizability of the findings. Next, the present observations were derived from a long-term cohort study conducted at a single medical center, with a relatively small sample size. As such, the relatively homogeneous ethnic background (northern Taiwanese population) may have introduced potential biases. Future studies with larger and more diverse populations across multiple centers might improve the robustness of the current findings. Concerning emphysema classification, this study used LAA% derived from HRCT scans, employing a threshold of 15% that aligns with local clinical practice guidelines. Despite following standardized HRCT procedures and involving two radiologists in each scan to minimize errors, differences in scan techniques and radiologist interpretations may have indirectly influenced LAA% outcomes, potentially affecting the data quality [61]. To minimize these biases, future work should implement consistent protocols and consider these factors in the analysis. Finally, although the mean decrease in the accuracy approach is straightforward to interpret, it may be susceptible to bias in the presence of correlated features. Future studies should consider integrating other permutation-based importance measures [62]. Utilizing multiple feature importance investigation methods may further provide a more robust assessment of feature contributions by mitigating correlation-related biases.

## 5. Conclusions

Despite pulmonary function tests and HRCT being considered primary tools for assessing emphysema progression, their high resource demands restrict timely evaluation, thereby impeding effective disease management and necessitating alternative approaches. Hence, in this study, we developed comprehensive ML models utilizing easily accessible data, including subjective (CAT scores) and objective (biomarkers and baseline characteristics) parameters. Moreover, the feasibility of integrating the *lnc-IL7R* advanced biomarker for emphysema classification (LAA% ≥15%) was evaluated. The feature importance analysis identified *lnc-IL7R* as the most influential predictor, followed by CAT scores and BMI. These findings suggest that the proposed models, which integrate readily obtainable variables, may serve as dependable and practical tools for classifying emphysema progression. This approach offers an efficient and less detrimental method for COPD management and may facilitate early monitoring in clinical settings with limited medical resources and personnel. To improve the applicability and robustness of the proposed model, future research should consider external validation with larger and more diverse populations. Additionally, integrating a permutation importance analysis may provide deeper insights and reduce potential biases arising from correlated variables.

## Figures and Tables

**Table 1:** Comparisons of the variations in characteristics between individuals grouped by a 15% percentage of the low-attenuation area (LAA%).

Variable	LAA% <15% (*n* = 46)	LAA% ≥15% (*n* = 34)	*p* Value
Age (years) ^a^	67.02 ± 7.65	68.44 ± 7.12	0.53
Gender (male/female) ^b^	44/2	30/4	0.39
BMI (kg/m^2^) ^a^	24.91 ± 4.27 **	21.24 ± 3.35 **	<0.01
Charlson comorbidity index (score) ^a^	4.13 ± 1.31	4.56 ± 2.08	0.71
CAT (score) ^a^	4.72 ± 3.31	6.24 ± 5.87	0.71
LAA (%) ^a^	8.75 ± 3.94 **	23.23 ± 7.13 **	<0.01
Comorbidities, *n* (%) ^b^			0.39
Cardiovascular disease	10 (21.73%)	6 (17.64%)	
Chronic heart failure	8 (17.39%)	4 (11.76%)	
Hypertension	15 (32.61%)	9 (26.47%)	
Metabolic syndrome	5 (10.86%)	2 (5.88%)	
Depression and anxiety	7 (15.21%)	5 (14.70%)	
Smoking status, *n* (%) ^b^			0.08
Current smoker	29 (63.04%)	16 (47.07%)	
Ex-smoker	15 (32.60%)	18 (52.94%)	
Never-smoker	2 (4.34%)	0	
Smoking pack years ^a^	53.5 ± 36.85	63.93 ± 35.34	0.11

Abbreviations: BMI, body mass index; CAT, Chronic Obstructive Pulmonary Disease Assessment Test. Data are expressed as the mean ± standard deviation. ^a^ Differences between the groups were assessed with the Mann−Whitney U-test. ^b^ Differences between the groups were assessed with Fisher’s exact test. ** p < 0.01.

**Table 2:** Comparisons of pulmonary function measures and biochemical details between individuals grouped by a 15% percentage of low-attenuation area (LAA%).

Variable	LAA% <15% (*n* = 46)	LAA% ≥15% (*n* = 34)	*p* Value
Pulmonary function details ^a^			
FEV_1_ (L)	1.71 ± 0.63 **	1.21 ± 0.48 **	<0.01
FEV_1_ (% predicted)	64.53 ± 19.87 **	50.25 ± 20.73 **	<0.01
FVC (% predicted)	84.47 ± 18.7	76.19 ± 23.78	0.09
FEV_1_/FVC (%)	60.03 ± 10.32 **	51.67 ± 10.96 **	<0.01
Biochemical details			
WBCs (10^3^/µL) ^b^	8.29 ± 3.01	8.08 ± 2.66	0.76
RBCs (10^6^/µL) ^b^	4.84 ± 0.67	4.67 ± 0.55	0.11
Platelets (10^3^/µL) ^a^	43.42 ± 4.73	42.84 ± 4.6	0.61
HCT (%) ^b^	225.47 ± 67.7	231.68 ± 64.84	0.24
Neutrophil count (µL) ^b^	5432.11 ± 2882.96	5339.77 ± 2365.39	0.98
Lymphocyte count (µL) ^b^	1900.87 ± 778.21	1804.91 ± 598.02	0.61
Eosinophil count (µL) ^b^	208.32 ± 142.42	214.61 ± 172.77	0.79
*lnc-IL7R* (fold) ^a^	0.57 ± 0.25 *	0.43 ± 0.22 *	0.01

Abbreviations: FEV_1_, forced expiratory volume in 1 s; FVC, forced vital capacity; WBCs, white blood cells; RBCs, red blood cells; HCT, hematocrit; *lnc-IL7R*, long non-coding interleukin-7 receptor α-subunit gene. Data are expressed as the mean ± standard deviation. ^a^ Differences between the groups were assessed with a Student’s *t-test*. ^b^ Differences between the groups were assessed with the Mann−Whitney U-test. * *p* < 0.05; ** *p* < 0.01. Note: the values of *lnc-IL7R* are expressed as fold changes relative to the normal control.

**Table 3:** Associations between biochemical details and the total lung low-attenuation area percentage (LAA%).

Variable	Total Lung LAA% (%)	
	Crude β Coefficient (95% CI) ^a^	Adjust β Coefficient (95% CI) ^b^
Biochemical details		
WBCs (10^3^/µL)	0.55 (−1.50 to 2.60)	0.33 (−1.46 to 2.12)
RBCs (10^6^/µL)	−0.94 (−2.95 to 1.08)	0.57 (−1.31 to 2.44)
Platelets (10^3^/µL)	0.39 (−1.66 to 2.44)	−0.61 (−2.45 to 1.26)
HCT (%)	−0.61 (−2.63 to 1.42)	1.59 (−0.37 to 3.54)
Neutrophil count (µL)	0.81 (−1.21 to 2.83)	0.41 (−1.39 to 2.2)
Lymphocyte count (µL)	−1.17 (−3.18 to 0.84)	−0.45 (−2.26 to 1.35)
Eosinophil count (µL)	−0.88 (−2.90 to 1.14)	0.31 (−1.52 to 2.13)
*lnc-IL7R* (fold)	−2.93 (−4.85 to −1.01) **	−2.65 (−4.33 to −0.97) **

Abbreviations: CI, confidence interval; WBCs, white blood cells; RBCs, red blood cells; HCT, hematocrit; *lnc-IL7R*, long non-coding interleukin-7 receptor α-subunit gene. ^a^ Simple logistic regression models. ^b^ Multivariable linear regression models were adjusted for age, sex, and body mass index. ** *p* < 0.01. Note: the expression values of *lnc-IL7R* are expressed as fold changes relative to the normal control.

**Table 4:** Comparisons of the cross-validation performance of the models established using multiple machine learning approaches.

Variable	Machine Learning Approach				
	LR	kNN	NB	SVM	RF
Accuracy (%)	65.62 ± 3.12	62.48 ± 7.82	73.59 ± 12.29	72.05 ± 7.45	75.15 ± 12.23
Precision (%)	61.9 ± 4.76	66.67 ± 47.14	74.1 ± 18.22	80.0 ± 17.89	72.5 ± 17.74
Recall (%)	38.46 ± 7.69	14.81 ± 13.86	63.33 ± 30.4	50.0 ± 20.0	66.67 ± 25.44
F1-score (%)	47.27 ± 7.27	23.33 ± 20.55	62.12 ± 22.83	57.0 ± 14.86	66.52 ± 19.31
AUROC (%)	74.09 ± 5.26	63.78 ± 8.39	75.6 ± 5.31	74.68 ± 8.84	78.31 ± 5.91

Abbreviations: LR, logistic regression; kNN, k-nearest neighbor; NB, naïve Bayes; SVM, support vector machine; RF, random forest; AUROC, area under the receiver operating characteristic curve.

**Table 5:** Comparison of the test performance of models established using multiple machine learning approaches based on bootstrapping techniques.

Variable	Machine Learning Approach				
	LR	kNN	NB	SVM	RF
Accuracy (%)	69.38 ± 12.98	62.76 ± 11.97	73.44 ± 1.56	72.42 ± 15.4	75.31 ± 11.12
Precision (%)	80.64 ± 18.88	31.84 ± 30.43	60.77 ± 0.77	63.06 ± 20.74	90.33 ± 10.36
Recall (%)	51.06 ± 19.68	20.40 ± 20.02	73.86 ± 1.14	70.83 ± 17.18	73.52 ± 13.70
F1-score (%)	60.63 ± 18.11	23.01 ± 20.73	66.67 ± 0.0	65.79 ± 16.88	80.19 ± 10.12
AUROC (%)	69.65 ± 12.07	51.19 ± 11.64	73.51 ± 20.78	72.92 ± 17.49	77.88 ± 11.52

Abbreviations: LR, logistic regression; kNN, k-nearest neighbor; NB, naïve Bayes; SVM, support vector machine; RF, random forest; AUROC, area under the receiver operating characteristic curve.

## Data Availability

All data were obtained from the Department of Thoracic Medicine, Taipei Medical University–Shuang Ho Hospital, between April 2015 and February 2021. Due to the presence of personal information, the dataset is not available in the Supplementary Materials. Requests for access to the dataset or related documents should be directed to the corresponding author.

## Supplementary Materials

The following supporting information can be downloaded at https://www.mdpi.com/article/10.3390/diagnostics15091165/s1.

## Abbreviations

AUROC, area under the receiver operating characteristic curve; BMI, body mass index; CAT, COPD Assessment Test; CI, confidence interval; COPD, chronic obstructive pulmonary disease; FEV_1_, forced expiratory volume in 1 s; FVC, forced vital capacity; HCT, hematocrit; HRCT, high-resolution computed tomography; kNN, k-nearest neighbor; LAA%, percentage of low attenuation area; *lnc-IL7R*, long non-coding interleukin-7 receptor α-subunit gene; LR, logistic regression; ML, machine learning; NB, naïve Bayes; PBMC, peripheral blood mononuclear cell; RBC, red blood cell; RF, random forest; SVM, support vector machine; WBC, white blood cell.
