# Supplementary material for: Leveraging Subjective Parameters and Biomarkers in Machine Learning Models: The Feasibility of lnc-IL7R for Managing Emphysema Progression
Source: Diagnostics (Basel). 2025 May 3;15(9):1165. doi: 10.3390/diagnostics15091165 (PMC12071574; doi:10.3390/diagnostics15091165)
Supplement: Supplementary file 1 [file diagnostics-15-01165-s001.zip › diagnostics-3574348-supplementary.pdf]

Supplementary Materials:

To examine whether BMI differences between emphysema severity groups (LAA% <15% vs. ≥15%) were independent of potential confounders, this study performed a multivariable logistic regression analysis adjusting for age, gender, and smoking status. The results indicated a significant association between BMI and emphysema severity (coefficient: –0.27; 95% CI: –0.42 to –0.11; $p < 0.01$), reinforcing that BMI was independently associated with group classification.

Additionally, to quantify the magnitude of the group difference in lnc-IL7R expression, Cohen’s d was calculated to evaluate the effect size. The analysis yielded a Cohen’s d of 0.68, indicating a moderate-to-large effect. This finding highlighted that the statistically significant difference in lnc-IL7R expression also represents a biologically meaningful distinction, further supporting its potential role in COPD severity classification.

Abbreviations

BMI, body-mass index; LAA%, percentage of low attenuation area; *lnc-IL7R*, long non-coding interleukin-7 receptor α-subunit gene.
